# Inner and outer setting factors that influence the implementation of the National Diabetes Prevention Program (National DPP) using the Consolidated Framework for Implementation Research (CFIR): a qualitative study

**DOI:** 10.1186/s43058-022-00350-x

**Published:** 2022-10-01

**Authors:** Lillian Madrigal, Olivia C. Manders, Michelle Kegler, Regine Haardörfer, Sarah Piper, Linelle M. Blais, Mary Beth Weber, Cam Escoffery

**Affiliations:** Rollins School of Public Health Emory University, 1518 Clifton Rd, Atlanta, GA 30322 USA

**Keywords:** CFIR, Qualitative analysis, Organizational factors, Inner setting, Outer setting, Diabetes prevention, Prediabetes, Enrollment, Scaling

## Abstract

**Background:**

Scaling evidence-based interventions are key to impacting population health. The National DPP lifestyle change program is one such intervention that has been scaled across the USA over the past 20 years; however, enrollment is an ongoing challenge. Furthermore, little is known about which organizations are most successful with program delivery, enrollment, and scaling. This study aims to understand more about the internal and external organization factors that impact program implementation and reach.

**Methods:**

Between August 2020 and January 2021, data were collected through semi-structured key informant interviews with 30 National DPP delivery organization implementers. This study uses a qualitative cross-case construct rating methodology to assess which Consolidated Framework for Implementation Research (CFIR) *inner* and *outer setting* constructs contributed (both in valence and magnitude) to the organization’s current level of implementation reach (measured by average participant enrollment per year). A construct by case matrix was created with ratings for each CFIR construct by interviewee and grouped by implementation reach level.

**Results:**

Across the 16 inner and outer setting constructs and subconstructs, the interviewees with greater enrollment per year provided stronger and more positive examples related to implementation and enrollment of the program, while the lower reach groups reported stronger and more negative examples across rated constructs. Four inner setting constructs/subconstructs (structural characteristics, compatibility, goals and feedback, and leadership engagement) were identified as “distinguishing” between enrollment reach levels based on the difference between groups by average rating, the examination of the number of extreme ratings within levels, and the thematic analysis of the content discussed. Within these constructs, factors such as organization size and administrative processes; program fit with existing organization services and programs; the presence of enrollment goals; and active leadership involvement in implementation were identified as influencing program reach.

**Conclusions:**

Our study identified a number of influential CFIR constructs and their impact on National DPP implementation reach. These findings can be leveraged to improve efforts in recruiting and assisting delivery organizations to increase the reach and scale of the National DPP as well as other evidence-based interventions.

**Supplementary Information:**

The online version contains supplementary material available at 10.1186/s43058-022-00350-x.

Contributions to the literature

- A large amount of resources have been dedicated to scaling up the CDC’s National DPP lifestyle change program; however, enrollment (reach) is an ongoing challenge.
- This is one of only a few implementation research studies of the National DPP focused on the organization level that use the CFIR construct rating qualitative methodology to explore the national implementation of this program.
- These results have broad application to understand how best to assist organizations to adopt, deliver, and scale evidence-based programs like the National DPP.
- This study builds upon CFIR research using this analysis approach and could facilitate comparisons across studies.

## Background

According to 2020 data, the Centers for Disease Control and Prevention (CDC) has reported that 96 million adults (38% of the adult population) in the USA have prediabetes, a condition that indicates a high risk, and progression to, type 2 diabetes [1]. The National DPP lifestyle change program is an evidence-based, year-long intervention with 22 sessions led by lifestyle coaches designed to prevent the progression to diabetes in people with prediabetes [2–4]. Since its inception in 2010 the National DPP has made great strides in raising awareness for and accessibility to its evidence-based lifestyle change program for people with prediabetes including establishing the program as a covered benefit for Medicare and Medicaid beneficiaries [3, 5]. However, program reach, the number or proportion of individuals participating in program, is lower than hoped. Approximately 2000 organizations of various types, sizes, and settings currently deliver the program across all 50 states and US territories [6, 7], but in 2017, the CDC reported that only 0.04% of the US adults with prediabetes had been reached in the first 4 years of the National DPP implementation [8, 9]. When efficacious programs (like the National DPP) can reach a large number of individuals, population impact occurs [10–13]. However, while organizations are adopting the program and expanding financial coverage for participants, enrollment or reach remains a challenge and a key focus for stakeholders [14–16].

Understanding factors related to adoption, implementation, and reach of the National DPP at the organizational level is critical to scaling the program. To date, research and evaluation of the National DPP has largely focused on participant-level outcomes [5, 8, 17]. These show the vast majority of participants are female (around 80%), 45 years or older, and with a prediabetes status determined by blood-based test [8, 18]. Other research about the National DPP have posited reasons for enrollment challenges including a lack of awareness in the prediabetes population of their diagnosis and/or the program, poor and underutilized referral systems, and challenges with setting up program reimbursement as possible barriers to enrollment [14–16, 19]. Organization-level evaluations have explored specific implementation strategies (referrals, partner networks, adaptation of materials, etc.) and have shown that use of incentives and healthcare provider-based referrals are promising practices to increase enrollment and participation [17, 20]. However, these studies focus on limited organizational characteristics, such as type and location (e.g., state), in their analyses.

In addition to intervention characteristics and program participants, other critical contextual factors internal and external to organizations may impact implementation outcomes. Chaudoir, Dugan, and Barr refer to these as organization (internal) and structural (external) level causal factors in their “Multi-Level Framework Predicting Implementation Outcomes” [21]. Furthermore, an understanding of organization characteristics (type, size, location, etc.) and factors within and surrounding an organization that influence the delivery of programs may be useful in developing strategies for recruiting new organizations and supporting current delivery. Thus, there is a need for an in-depth and rigorous examination of internal and external organization factors and the ways they impact the implementation success of the National DPP.

This study aims to fill this gap by applying the Consolidated Framework for Implementation Research (CFIR), a metatheory comprised of constructs associated with implementation, that has been increasingly utilized in public health research to understand diverse aspects of implementation processes and outcomes [22–24]. Within diabetes prevention and management, researchers most commonly have used CFIR to examine facilitators and barriers to program implementation [25–27]. For example, Wilcox et al. (2020) used CFIR to identify predictive constructs with implementation outcomes for a cultural adaptation of the National DPP for African-American Churches in the South [28]. To our knowledge, CFIR has not been used to examine inner and outer setting factors related to enrollment and implementation in the National DPP across organizations.

Two of the five domains, the *inner setting* and *outer setting,* focus on internal and external organization factors [23]. The CFIR constructs listed in the *inner setting* domain aim to capture the complexity within the organization related to implementation (e.g., structural characteristics, culture, and readiness for implementation). The *outer setting* constructs provide insight into the greater environments and external context which constrain organizations or facilitate their ability to carry out the intervention (e.g., cosmopolitanism, peer pressure, and external policies and incentives).

To contribute to the current knowledge of the National DPP, this study will explore factors related to the internal and external organization (operationalized through the CFIR *inner* and *outer setting* constructs) to understand relationships between these constructs and program implementation and enrollment (reach). Insights gained can inform strategies to expand the capacity of delivery organizations to increase engagement in the program and scale the program up and out nationally [5, 15, 16].

## Methods

In 2019, Emory Center’s Diabetes Technical Assistance and Training Center (DTTAC) was funded to study the role of Lifestyle Coaches in the implementation of the National DPP through the CDC’s Division of Diabetes Translation’s Innovations to Grow Enrollment and Retention (InGEAR) project. Over the last 10 years, DTTAC has directly trained over 5000 lifestyle coaches representing over 2000 organizations across all 50 states. The National DPP implementers included in this study participated in Emory’s DTTAC Lifestyle Coach and/or Master Trainer Select training programs and/or subscribed to the center’s resources.

This study uses a qualitative cross-case construct rating methodology to assess which CFIR constructs contributed both in valence (positive or negative influence) and magnitude (combined influence) to the organization’s current level of implementation reach (measured by average participant enrollment per year). Between August 2020 and January 2021, data were collected through semi-structured key informant interviews with 30 National DPP delivery organization implementers (see sampling for selection criteria and procedures). This study was reviewed and determined to be exempt by the Emory University Institutional Review Board (STUDY00000658).

### Sampling

DTTAC provided a list of National DPP implementers (*n* = 239) and their basic organization characteristics (organization type, location, level of implementation, etc.) that were generated from a call for study participation via the DTTAC mailing list and newsletter. Potential participants were stratified into groups of higher (> 85 program participants), medium (26–85 program participants), and lower (≤ 25 program participants) reach organizations based on total enrollment to date. Participant enrollment data from the CDC and DTTAC’s records were used to create the higher, medium, and lower tertile ranges.

We purposively selected participants to reflect the diversity of implementers by organization type, length of program delivery, urbanicity, populations served, and size (Table 2). Due to variations in these organization characteristics, we planned to interview at least 30 participants. Thirty-nine National DPP implementers across the three organization implementation levels were selected; nine either did not respond to the invitation or declined to participate. The final sample included 30 National DPP organization key informants located in 24 states and territories. During the analysis, we found that after conducting the 30 interviews we had reached saturation or the point at which no new information relating to the CFIR constructs was identified in each of the groups. Organization staff reported a range of 5 to 600 enrolled participants to date. In the analysis, to control for length of delivery, enrollment numbers were divided by years of delivery to produce the average enrollment per year for each organization. The interviewees were re-stratified by enrolled participants per year into high (36–150), medium (17–35), and low (5–16) reach levels (Table 2).

### Instruments

We developed a semi-structured interview guide with questions adapted from the CFIR guide (CFIR Research Team) and studies using similar methods [29, 30]. Open-ended question topics included interviewee training and background, program implementation success in terms of reach and sustainability, and 16 questions with suggested probes for each of the inner and outer setting CFIR constructs and sub-constructs (see Additional Table 1 for interview guide). Questions were posed in a way to encourage discussion about *how* each construct or subconstruct has positively or negatively impacted program implementation, particularly related to the outcome of reach (enrollment numbers). For example, the culture construct question asked, “In what ways do you think your organization's culture (general beliefs, values, assumptions that people embrace) affect the implementation of the National DPP?” The probe following asked, “How does the organization’s culture impact enrollment of participants in particular?” The interview guide was reviewed by National DPP subject matter experts and pre-tested.

### Data collection

Interviewees were invited to participate in a 60-min interview using Emory’s secure Zoom videoconferencing account; verbal consent and permission to audio-record were obtained prior to initiating the interview. Interviews were conducted from August 2020 to January 2021. *A*ll recordings were transcribed by a third party, quality-checked, deidentified, and uploaded into MAXQDA 2020 [31] for coding and analysis.

### Interviewee descriptive statistics

Interviewee organizations were categorized into one of five organization types: healthcare/hospitals, community-based healthcare (community health centers, federally qualified health centers, Indian Health Service, etc.) community-based organizations (YMCAs, local nonprofits, etc.), government agencies (state/local health departments), and other (health plans, insurers, worksite wellness programs, universities, private businesses). Descriptive statistics were run on organization characteristics including years delivering the National DPP, size based on the approximate number of people served across the entire organization annually, CDC Diabetes Prevention Recognition Program (DPRP) status^1^ for the National DPP (Full vs Pending/Preliminary status), location of the organization by US region, and race/ethnicity of the National DPP participant population at the organization (Table 2).

### Coding interviews

A deductive codebook of CFIR constructs and interview questions was developed; in vivo (inductive) codes were added as relevant topics were identified during initial coding. The codebook was tested for clarity and relevancy and refined prior to coding. Coders (LM and OM) independently coded each transcript and conducted intercoder agreement, discussing and reaching consensus where there were discrepancies in coding. Double coding and intercoder agreement was performed on one third of the transcripts (*n* = 10) to ensure intercoder reliability [32, 33].

### Construct rating

We used a qualitative construct rating analysis approach from Damschroder and Lowery (2013) to rate CFIR constructs related to implementation outcomes [30]. Applying this methodology, we identified distinguishing constructs among organizations with different levels of implementation reach and identified themes within those constructs that contribute to those differences.

Coded segments for each CFIR construct were exported, sorted by organization, grouped by implementation reach level, and reviewed independently by both analysts (LM and OM). All segments were assessed by construct for valence (positive or negative influence on implementation) using construct rating criteria (Table 1). Segments were scored with a − 2 to + 2, a 5-point bi-polar scale; where there was more than one segment for an interview, the analysts discussed each segment and assigned an average score. Interviews that had positive statements about a construct’s influence on implementation were scored with a 1 or 2 depending on the level of detail and impact on enrollment. Likewise, interviews with negative examples were scored with a − 1 or − 2. An equal mix of positive and negative influences received a score of zero.

**Table 1:** Rating criteria

**Rating**	**Criteria**
− 2	Participant describes with detail how the construct is a negative/impeding influence on implementation related particularly to participant enrollment
− 1	Participant makes statements about the construct as a negative influence/impeding influence on implementation generally
0	Mixed—participant describes both positive and negative statements about the construct in regards to general implementation and/or enrollment
1	Participant makes statements about the construct as a positive influence/facilitating influence on implementation generally
2	Participant describes with detail how the construct is a positive influence/facilitating influence on implementation related particularly to participant enrollment
X	Purely descriptive, no impact upon implementation or enrollment was described
M	Construct was not discussed during the interview

Coders met weekly to discuss ratings and consensual validation was achieved through a process of deliberation and consensus. Since our sample had only one interviewee per organization, we adapted Damschroder and Lowery’s methods to remove the synthesis of findings among multiple interviewees at the organization level. Once all transcripts were rated across all 16 CFIR constructs and subconstructs (a case-oriented approach, because ratings were applied within each case), both coders examined them across cases by construct (the variable-oriented approach, since each construct is compared across cases).

### Analysis and Interpretation

**Fig. 1:**
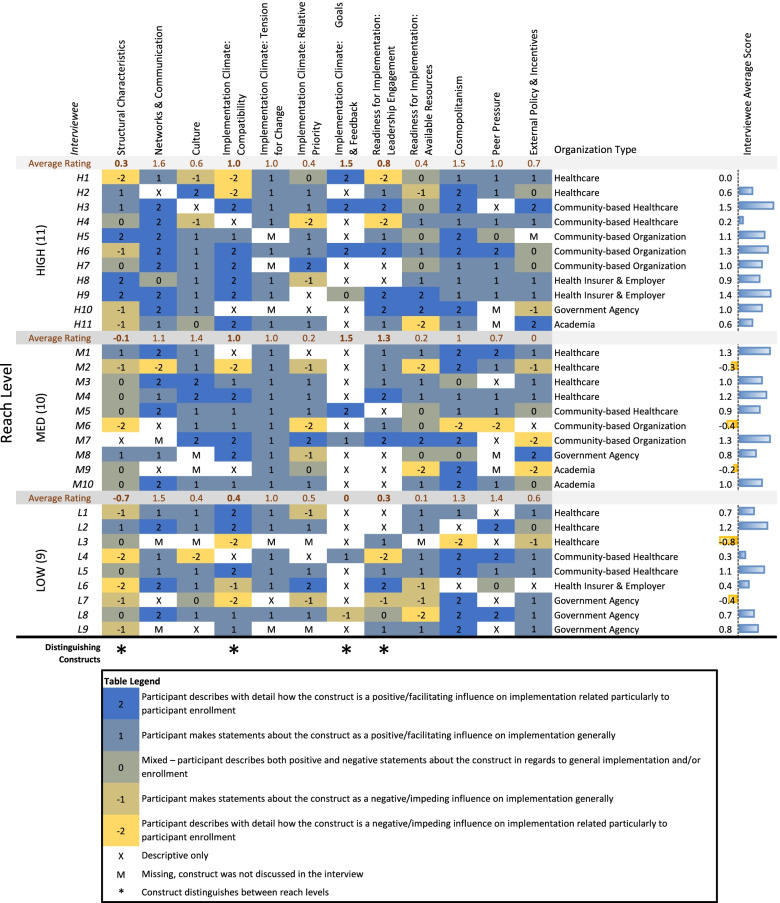
Construct rating matrix

A construct by case matrix was created that listed the ratings for each CFIR construct by interviewee and grouped by implementation reach level (Fig. 1). This stage of the analysis focused on discerning patterns across the high, medium, and low implementation reach groups. Average rating scores were calculated for each construct by reach level to identify patterns. Interviewees within each reach level were also sorted by organization type and given a summative average rating across all of the constructs to more easily identify rating differences.

Positive and negative extremes were discussed across all constructs and interviewees at every level. Constructs/subconstructs were identified as *distinguishing* based on the difference between groups by average rating (generally if the difference was over ± 0.5 between levels), the examination of the number of extreme (+ 2 or − 2) ratings within levels, and through thematic analysis based on the content discussed.

## Results

### Organization characteristics

Program staff from 30 unique implementing organizations were interviewed. CDC-recognized National DPP organizations designate Program Coordinators who supervise daily operations of the program, provide guidance and support to lifestyle coaches, and monitor and submit all program data to the CDC. Of these, all but one were Program Coordinators and 26 served in a combination of roles as Program Coordinators, Lifestyle Coaches, and/or Master Trainers for the National DPP.

About one third (9) of interviewees were from healthcare or hospital settings, followed by a near equal number from community-based healthcare (5, 17%), and other types of organizations (6, 20%) (Table 2). The majority of organizations were in the initial (13, 43%) or intermediate (13, 43%) phases of delivery and held Pending/Preliminary CDC DPRP status (20, 67%). There was consistent representation across the organization size categories and geographic regions. The vast majority have National DPP participants from White (80%), Black (70%), and Hispanic/Latino (63%) racial and ethnic backgrounds. Fewer organizations reported serving Alaska Native/Native American (27%) and Asian/Pacific Islander (27%) participants in their programs.

**Table 2:** Interviewee organization characteristics by implementation reach

	Implementation reach			
*Implementation level based on reach calculated as the average number of participants enrolled per year*	Low *5–16/year*	Med *17–35/year*	High *36–150/year*	Total (%)
*Number of interviewees*	9	10	11	30
Organization type (*n*)				
Healthcare/hospitals	3	4	2	9 (30%)
Community-based healthcare	2	1	2	5 (17%)
Community-based organizations	-	2	3	5 (17%)
Government agencies	3	1	1	5 (17%)
Other: health insurers, employers, academia	1	2	2	6 (20%)
Years delivering the National DPP (*n*)				
0–2 years: initial delivery phase	6	5	2	13 (43%)
3–4 years: intermediate delivery phase	2	5	6	13 (43%)
5 + years: long-term delivery phase	1	-	3	4 (13%)
Organization size (*n*)				
0–1000 people served annually across all services and programs	1	3	1	5 (17%)
1001–10,000 people	4	5	2	11 (37%)
10,001–100,000 people	4	1	2	7 (23%)
Over 100,000 people	-	1	5	6 (20%)
*Missing*			1	1 (3%)
CDC DPRP recognition status^a^ (*n*)				
Pending/preliminary	7	8	5	20 (67%)
Fully recognized	2	2	6	10 (33%)
Geographic region in the USA (*n*)				
Northeast	3	1	2	6 (20%)
Southeast	3	4	3	10 (33%)
Midwest	-	1	3	4 (13%)
Southwest	3	2	1	6 (20%)
West	-	1	2	3 (10%)
Other (US territories)	-	1	-	1 (3%)
Populations served (*n*)				
White/Caucasian	7	8	9	24 (80%)
Black/African-American	7	5	9	21 (70%)
Hispanic/Latino	7	4	8	19 (63%)
Alaska Native/American Indian	3	-	5	8 (27%)
Pacific Islander/Asian	2	2	4	8 (27%)

^a^For more details on CDC’s DPRP recognition status requirements see https://www.cdc.gov/diabetes/prevention/requirements-recognition.htm

Representation across levels of implementation reach by organization type was also fairly consistent. In terms of years delivering the program, those in the higher reach group had delivered the program for the longest number of years, while the medium and lower reach groups included many organizations in the initial delivery phase (0–2 years). The higher reach group also tended to include those from larger organizations and more often with full DPRP recognition status.

### CFIR construct findings

The construct rating matrix provides the CFIR ratings for each inner and outer setting construct by organization interviewee grouped by implementation reach level (Fig. 1). Overall, the majority of interviewees were net positive in terms of their implementation examples across all of the constructs (Fig. 1 interviewee average score). However, the interviewees in the higher reach group provided stronger (+ 2) and more instances of positive examples across all constructs related to implementation and enrollment, while the low reach group stronger (− 2) and more instances of negative examples across all rated constructs. Four constructs/subconstructs (incentives and rewards, learning climate, access to knowledge and information, and patient needs and resources) were not discussed in relation to implementation reach sufficiently in the interviews to conduct the construct rating and were omitted from the matrix. The following four *inner setting* constructs/subconstructs were identified as distinguishing: structural characteristics, compatibility, goals and feedback, and leadership engagement. No *outer setting* constructs were distinguishing. The following results will highlight these constructs with a discussion of the thematic analysis of the coded segments and supporting quotes.

#### The structural characteristics

The structural characteristics *construct is comprised of many traditional measures of context and organization characteristics (organization size, type, location, *etc*.).* Among the interviewees, *Structural Characteristics* often involved discussions of organization infrastructure for the program (physical space, staff size, etc.). As this construct contains a multitude of dimensions, interviewees frequently described both positive and negative examples, resulting in many mixed ratings. This construct appears to distinguish the high and medium reach level organizations from the low reach group. In the medium and high reach levels, interviewees often discussed both the benefits and challenges of implementation related to structural characteristics. For example, one interviewee commented on how the size of the organization can both help and hinder National DPP implementation (exemplary quotes provided in Table 3).

**Table 3:** Distinguishing construct themes and exemplary quotes

**Construct themes**	**Positive/mixed quote**	**Negative quote**
Structural characteristics: comprised of many traditional measures of context and organization characteristics (organization size, type, location, etc.)		
High and medium reach organizations described both positive and negative examples related to implementation, while low reach organizations discussed mostly negative examples	“so, we’re a pretty big organization. […] There are pros and cons to everything. So I think our size is a pro just because we have a large population, like a patient population, in which to draw from. […] One thing that can make it a barrier, though, as far as trying to get referrals and spread the word is when it’s a huge organization and there’s a lot going on, sometimes it is hard to get the message across when there’s just so much other stuff going on.” Interviewee M2 Medium reach Healthcare	“[…] for us to start applying for a grant, we somewhere in our process have to involve the city council. And in addition to that process once the city council okays on us applying for the grant, we receive the grant. Now we have to implement that grant into the city’s budget. So that becomes really tedious and becomes a really huge pain as opposed to a nonprofit.” Interviewee L9 Low reach Government agency
Compatibility: a subconstruct of implementation climate and relates to how the intervention fits within the organization and its existing workflows, systems, and services		
High and medium reach organizations described how the National DPP was complementary to similar services and programs, had systems that could easily integrate the National DPP, and overall better fit compared to medium and low reach organizations	“[…] for example if we see a patient who’s interested […] the most common one of course is DPP and then the diabetes health management. […] ‘And hey, you have diabetes, it runs in your family, well this program would be better for you and vice versa. Oh, well you have diabetes, we’d love to have you, you’d still benefit but, this one might be a little bit more appropriate for you.’ But I think it’s a nice complement and it nicely rounds out the services that we offer.” Interviewee H8 High reach Health insurer	“We have to force it to fit…I feel like it needs to be a part of the entire process, like if someone’s coming in for one particular service they should be screened for being at risk of having Type 2 diabetes. And we've done it, but it's only been during specific times and then it goes away…So I would love to see it more integrated into all of the programs.” Interviewee L7 Low reach Government agency
Goals and feedback: a subconstruct of implementation climate and refers to the degree to which goals are clearly communicated, acted upon, and fed back to staff, as well as the alignment of that feedback with goals		
High reach organizations discussed formal enrollment goals and provided feedback to team around goals, while medium and low reach organizations tended to have no formal goals set	“[…] we always have a goal an enrollment goal. So we always reach the goal and we have a waiting list. There’s always a waiting list and as I said, that’s something that we’re very proud of. […] it’s nice to have the number, I like numbers. Tell me what you want, I'll go for that number.” Interviewee H3 High reach Community-based healthcare	“It’s so new that I don’t think that they would even know where to begin […] we have this great program that’s been developed by the CDC and we're implementing it, but until we kind of establish a firmer way of recruitment, they’re not too hardcore.” Interviewee L6 Low reach Health insurer and employer
Leadership engagement: a subconstruct of readiness for implementation and refers to the commitment, involvement, and accountability of leaders and managers with the implementation of the program		
High reach organizations included strong examples of leadership engagement leading to increased enrollment/scaling. Lower reach group described leadership as not being engaged enough; not doing enough to understand the program, and taking a “hands off” approach	“Our leadership has been great […] I'll just give an example when we were going to be Medicare suppliers or applying for Medicare reimbursement. […] And because we had physicians on our board and people that knew about the program, they knew about the process even with Medicare we really had buy-in there because they were able to explain it […] So they really came together and got everybody on board and we were able to get those numbers and submit the application.” Interviewee H6 High reach Community-based organization	“[…] I think leadership – just to actually sit down and know – understand the program a little bit better and understanding the goals that are attached to it and understanding the work that’s needed to get done. That’s it. I think that’s where the gap comes in.” Interviewee L8 Low reach Government agency

However, among the low reach group, the vast majority of the coded *structural characteristics* segments were rated negatively. These interviewees reported difficulties with limited infrastructure for the program, lack of staff and staff time, challenges with developing referral systems, and administrative/bureaucratic hurdles due to their organization type. For example, an interviewee from a local government agency shared many challenges involved with applying for and implementing grants due to their organization structure and bureaucracy. Across all cases, organization type and how it impacted organization reach to populations, available infrastructure/resources, administrative processes, and reputation in the community was most salient.

#### Compatibility

Compatibility is *a subconstruct of Implementation Climate and relates to how the intervention fits within the organization and its existing workflows, systems, and services.* High and medium reach groups more often yielded strong positive examples of *compatibility* impacting implementation, compared to those in the low reach group. Interviewees describing positive examples of the influence of *compatibility* on implementation often mentioned that their organization offered complementary programs to the National DPP (e.g., diabetes self-management, nutrition education, and fitness classes). This allowed them to more easily adopt and implement the National DPP. As described by one high reach organization interviewee, “it’s a nice complement and it nicely rounds out the services that we offer.”

In the strongest positive examples, interviewees shared how other programs within their organization referred program participants to the National DPP and vice versa. They also gave positive examples of how the National DPP was embedded in their workflows and systems via the electronic health records (EHR) or other referral processes, all of which support enrollment efforts. Two high reach group interviewees described challenges introducing the National DPP into their organization systems, but by taking time to educate key leaders and staff about the program, they were able to overcome those challenges and succeed with implementation. Conversely, in strong negative examples of *compatibility*, interviewees struggling to implement the National DPP described how it was different from the typical services and programs provided by their organization and was not embedded into their current systems, one interviewee described this as having to “force it to fit.”

Across all reach levels, there were some additional themes related to *compatibility.* A commonly voiced complaint was how time-consuming and burdensome the data reporting to the CDC DPRP is compared to other evidence-based interventions implemented at their organizations. Lastly, in a few cases, interviewees shared that their organization had a large number of chronic disease programs, and this created challenges for staff to remember to refer to the National DPP.

#### Goals and feedback

Goals and feedback *is a subconstruct of Implementation Climate and refers to the degree to which goals are clearly communicated, acted upon, and fed back to staff, as well as the alignment of that feedback with goals.* We asked interviewees to discuss how enrollment goals (target number of participants to recruit each year) set by them or leaders at their organization impacted their implementation efforts. Overall, interviewees did not provide many details on how enrollment goals impact implementation (hence many interviewees have an “X” on the matrix for this construct). However, while interviewees lacked examples for the construct rating, this construct was distinguishing among reach groups by some of the extreme examples in the higher reach group and the thematic analysis which found the presence or absence of enrollment goals differed by reach level. The majority of the high implementation organizations (*n* = 9, 82%) had formal enrollment goals set by organization leadership or the program coordinator. In comparison, only four (40%) of the medium and three (33%) low reach interviewees reported having enrollment goals.

When organizations had enrollment goals there were clear examples as to how goals facilitated enrollment. One high reach group interviewee described how goals motivate the staff to increase their referrals and enrollment, saying, “[…] it’s nice to have the number, I like numbers. Tell me what you want, I’ll go for that number.” For organizations that did not have formal enrollment goals, interviewees mentioned other goals such as achieving CDC DPRP recognition status, billing Centers for Medicare & Medicaid Services (CMS)/becoming a Medicare DPP supplier, training their staff to implement the program, general diabetes prevention in their communities, or focusing on the retention of their current cohorts first before attempting to enroll more participants. One interviewee from a medium reach organization said because their focus is on establishing a process for billing CMS, they are not concerned about enrollment and prefer a small cohort at the moment. Multiple interviewees that currently did not have enrollment goals said they were interested in setting formal enrollment goals. While the interviews focused on pre-COVID-19 implementation, a few interviewees mentioned how COVID-19 had disrupted their implementation and therefore currently enrollment goals were not a priority.

#### Leadership engagement

Leadership engagement is *a subconstruct of Readiness for Implementation and refers to the commitment, involvement, and accountability of leaders and managers with the implementation of the program.* This construct appeared numerous times throughout most interviews. While the majority of interviewees simply said they have “support” from their leadership, when asked to describe this support in terms of *leadership engagement* their examples varied greatly. Examples included: leadership being aware of all program activities and events, making presentations to promote the program, connecting with other organization leaders/partners for the program, facilitating internal organization processes (e.g., board approvals, system establishment) for the program, obtaining resources including adequate staffing for the program, and providing the program for free to organization employees.

*Leadership engagement* was a distinguishing construct as high and medium reach cases had more strong positive examples of *leadership engagement* compared to the low reach cases. High and medium reach interviewees also more often connected *leadership engagement* with positive examples of successful enrollment efforts and growing the infrastructure for the program. The lower reach group had more mixed experiences with this construct. Leadership was described as not being engaged enough; not doing enough to understand the program, and taking a “hands off” approach. The key message from all interviewees across reach levels was that *leadership engagement* is highly desired and appreciated when available. Leadership support and knowledge of the program was discussed as a strong facilitator in implementing and scaling the program.

## Discussion

This study applied CFIR to examine the internal and external organization factors influencing National DPP implementation. The four distinguishing constructs from the inner setting (structural characteristics, compatibility, goals & feedback, and leadership engagement) indicate that there are multiple factors internal to the organization that can impact implementation and enrollment success. Our findings are consistent with other studies that have found that some of these same constructs influence successful implementation—particularly within the inner setting domains such as leadership engagement and the implementation climate subconstructs [29, 30, 34, 35].

Similar to previous research, positive *leadership engagement* on implementation involved going beyond surface-level support of the program and was highlighted by taking an active role in understanding the program, attending program events, promoting the program, and providing resources [29, 30, 34]. The distinguishing implementation climate subconstructs of *compatibility*,* relative priority*, and *goals and feedback* indicated that for organizations at higher reach levels, the National DPP fit better with existing services, health promotion programs, and systems, was prioritized by the organization leadership, and had formal enrollment goals outlined.

To date, there is a lack of evaluation of the National DPP using CFIR constructs; however, other lifestyle change programs also focused on physical activity and nourishment behaviors to achieve weight loss have been studied using CFIR [30, 36–38]. These studies have also found a heavy emphasis on the inner setting when exploring program implementation successes and challenges. Related the outer setting, their results described challenges with billing and program reimbursement/financing policies. Our study did not find any outer setting constructs distinguishing between reach levels; however, there were notable themes that emerged from the data around the importance of external partnerships, understanding participant needs, benefits of learning from and competing with other National DPP delivery organizations, and challenges with reimbursement programs (like Medicare DPP). Likewise, the constructs that were not rated (incentives and rewards, learning climate, access to knowledge & information, and patient needs & resources), also provided other insights into the program, such as examples of program participant barriers and challenges, however they did not talk in enough detail about these influences on implementation and enrollment to be included in this analysis.

The findings of this study have the potential to facilitate implementation of the National DPP. While the National DPP provides guidance on the standard infrastructure needed for organizations to deliver the program (*structural characteristics*) and the importance of partnerships (*cosmopolitanism*), previously there has been less of a conversation about organization compatibility, priorities, goal setting and feedback, and active leadership engagement which we identified as important in this study [39, 40]. The CDC should consider inclusion of CFIR-related constructs such as leadership engagement in the CDC DPRP Organizational Capacity Assessment, a suggested tool that delivery organizations use at the time of adoption. The assessment is primarily focused on the minimum requirements to deliver the program (e.g., classroom space, equipment, staff requirements) but does not help identify which organization characteristics may be particularly suited to reach a large number of participants and successfully scale. The current version does include about many of the CFIR constructs including the ones identified as distinguishing in our study. For example, there is no discussion of how well the program “fits” within an organization’s current programs and services or how the program would be prioritized if implemented. Inclusion of these constructs could assist with identifying key gaps in organization adoption readiness, enrollment, and scalability [41, 42].

To support low reach delivery organizations, the CDC and other National DPP technical assistance providers should consider guidance and resources to increase program compatibility, prioritization, goal setting, and leadership engagement. However, the main program implementers may not have control over these conditions. For example, a frequently mentioned challenge in the relative priority construct was limited staff and staff time dedicated to the program. Organizations may require more assistance and resources from the CDC and others to ensure adequate staff are not only hired and properly trained, but that their time is dedicated sufficiently to the National DPP. These considerations may also be applicable to other evidence-based programs as well.

### Future research

More research is required to understand how internal and external organization factors influence implementation in order to continue to scale the National DPP. While this study did not identify any outer setting constructs as distinguishing, the outer setting *c*onstruct, *cosmopolitanism*, has appeared in National DPP studies focused on referrals from providers, health systems, and other community partners [17, 20, 40]. Organizations with strong external partnerships were able to leverage this into increased referrals to their programs. Our study may not have found outer setting constructs as distinguishing because the interviewees focused more heavily on the inner setting constructs and interviewees reported mostly neutral and positive experiences regardless of reach level (Fig. 1). Additional research may be warranted to explore how the outer setting may affect the National DPP or other chronic disease programs. Likewise, future research should also include exploration of the other CFIR domains (intervention characteristics, process, and characteristics of individuals) to provide a holistic perspective on factors related to reach.

It is challenging to compare the National DPP to other programs using CFIR, as researchers have focused on different dimensions of CFIR constructs, often times based on relevant factors for the specific program. For example in the Cannon et al. 2019 study, the *culture* construct was operationalized as implementation of staff turnover [43], whereas in our study we focused on an organization’s general beliefs, values, and assumptions. This is a shortcoming of CFIR itself which has been criticized as very complex and multi-dimensional, and requiring more nuanced detail [44]. The developers of CFIR have developed a second version of the framework with the goal of addressing this criticism and other gaps that may be helpful to use in subsequent studies [45].

Measuring CFIR constructs quantitatively is a growing area that has great potential to assist with understanding the relationship between implementation factors and outcomes [46, 47]. Implementation science researchers have started testing quantitative measures for CFIR constructs; however, more work is needed in this area to fully understand the validity and reliability of these constructs, how they are operationalized in practice, and their associations with implementation outcomes [48–52]. CFIR quantitative measures have typically examined relationships between constructs and shorter-term implementation (e.g., adoption) rather than later-term outcomes like sustainability [21]. Using CFIR measures across the continuum of implementation will be critical to assess differences in key factors related to outcomes in the pre-adoption, early adoption, and maintenance phases [53].

### Strengths and limitations

Strengths of this study include a priori use of CFIR constructs and instruments, highly trained qualitative researchers and coders, rigorous double coding and analysis of data, and the application of the construct rating methods employed by other researchers. However, this study had several limitations that should be considered. First, there are 2000 + organizations delivering this program nationwide, and our study included only 30 in our sample, therefore results may be limited. In addition, we recruited from Emory’s DTTAC contact list and while this population is very large and diverse, there may be implementation differences between this group and the larger National DPP population of implementers because of the training and technical assistance they receive from Emory.

Second, only one interview was conducted per organization. Other papers using this construct rating method typically include 2–3 interviewees per organization [29, 30]. However, we were able to talk with 30 different organizations which is a higher number of unique organizations than is typical for this analysis. Instead of depth within organizations, we focused on breadth across a diverse range of organizations and focused on the best possible informant to answer our questions (the Program Coordinator). In order to capture the diversity in National DPP implementation, we did not limit organizations based on a specific number of years of delivery for recruitment. Instead, we operationalized reach using the average enrollment per year of delivery. Reach numbers were self-reported by the interviewee and we did not have access to the programmatic data to confirm or examine changes in enrollment over the years of delivery. While imperfect, using the average enrollment per year helps compare implementation success across organizations.

For this study we only focused on the inner and outer setting constructs of CFIR and decidedly focused on the organization-level perspective, which limits our understanding of the other dimensions of implementation (intervention characteristics, process, and characteristics of individuals). Lastly, in early 2020 the COVID-19 pandemic disrupted the National DPP, a largely in-person program, greatly. We did try to limit our interview discussions to pre-COVID implementation and saved COVID-19 related conversations to the end of the interview. As the pandemic has been an unignorable outer setting/external factor it is hard to say how much of the discussion of these topics was impacted by implementers who found themselves in a “survival/adaptation mode” at the time of the interview. The recently released CFIR 2.0 update now includes a new *outer setting* construct named “*critical incidents*” to capture “large-scale and/or unanticipated events” that may provide useful for situations such as these [54].

## Conclusions

This study found that there are a number of CFIR inner setting constructs that impact implementation reach of the National DPP. This understanding can be leveraged to improve efforts in recruiting and assisting delivery organizations to increase the reach and scale of the program. This is one of only a few studies of the National DPP at the organization level and to use the CFIR construct rating qualitative methodology to explore the national implementation of this program. More focused attention to program compatibility, prioritization, setting program goals, and leadership engagement has the potential to improve program implementation. Furthermore, these results have broader application to understand how best to assist organizations to adopt, deliver, and scale evidence-based programs.

## Supplementary Information

**Additional file 1: Table 1.** Interview Guide Questions.

## Data Availability

De-identified qualitative data is available by reasonable request to the corresponding author. Quantitative data was from DTTAC databases which are not publicly available.

## Abbreviations

CDC: Centers for Disease Control and Prevention
CFIR: Consolidated Framework for Implementation Research
CMS: Centers for Medicare & Medicaid Services
DPP: Diabetes Prevention Program
DPRP: Diabetes Prevention Recognition Program
DTTAC: Diabetes Training and Technical Assistance Center
EHR: Electronic Health Record
InGEAR: Innovations to Grow Enrollment and Retention
MDPP: Medicare Diabetes Prevention Program

## Footnotes

1 The DPRP provides national quality standards to ensure organizations are delivering the program with fidelity. These standards and procedures are updated every three years based on new dietary, physical activity, self-efficacy, delivery modality, and other type 2 diabetes prevention evidence. CDC-recognized organizations work toward progressing from pending to preliminary to full recognition status.
