# Supplementary material for: Inner and outer setting factors that influence the implementation of the National Diabetes Prevention Program (National DPP) using the Consolidated Framework for Implementation Research (CFIR): a qualitative study
Source: Implement Sci Commun. 2022 Oct 1;3:104. doi: 10.1186/s43058-022-00350-x (PMC9526531; doi:10.1186/s43058-022-00350-x)
Supplement: Supplementary file 1 — Additional file 1: Table 1. Interview Guide Questions. [file 43058_2022_350_MOESM1_ESM.docx]

**Additional Table 1 Interview Guide Questions**

| **Topic/Construct** | **Example questions** |
| --- | --- |
| *Background* | Can you tell us a little about your organization’s history with the National DPP lifestyle change program? [Learn about adoption of the intervention] |
| *Outcomes - Reach* | How would you define success when it comes to your organization’s implementation of the National DPP?  How successful do you think your organization has been based on your definition of success? |
| *Outcome - Sustainability* | How would you define sustainability for your organizations as it pertains to delivering the National DPP? |
| *Inner Setting* | |
| Structural Characteristics | CFIR Guide Question: How will the infrastructure of your organization (social architecture, age, maturity, size, or physical layout) affect the implementation of the intervention?  Adapted Question: How do you think aspects of infrastructure (size, physical space, staff size, age of the organization, type of organization, etc.) impact your ability to implement the National DPP lifestyle change program at your organization? |
| Networks & Communications | CFIR Guide Questions: Can you describe your working relationships with your colleagues? Do you meet (formally or informally) with a team of people? Are meetings, such as staff meetings, held regularly?  Adapted Question: Describe the internal communication processes within your organization that facilitate the implementation of the National DPP lifestyle change program? |
| Culture | CFIR Guide Question: How do you think your organization's culture (general beliefs, values, assumptions that people embrace) will affect the implementation of the intervention?  Adapted Question: In what ways do you think your organization's culture (general beliefs, values, assumptions that people embrace) affect the implementation of the National DPP? Can you describe an example that highlights this? |
| Implementation Climate - Compatibility | CFIR Guide Question: How well does the intervention fit with existing work processes and practices in your setting?  Adapted Question: How well does the National DPP fit with existing work processes and practices in your setting? |
| Implementation Climate - Tension for Change | CFIR Guide Question: Is there a strong need for this intervention?  Adapted Question: Would you say there is a strong need for the National DPP at your organization? Why/Why not |
| Implementation Climate - Relative Priority | CFIR Guide Questions: What kinds of high-priority initiatives or activities are already happening in your setting? What is the priority of getting the intervention implemented relative to other initiatives that are happening now?  Adapted Questions: What would you say are the key priority programs/activities at your organization currently? Where is the National DPP/diabetes prevention at on the priority list? |
| Implementation Climate - Organizational incentives & rewards | CFIR Guide Question: What kinds of incentives are there to help ensure that the implementation of the intervention is successful?  Adapted Question: What kinds of incentives are in place at your organization to help ensure that the implementation of the National DPP is successful? |
| Implementation Climate - Goals and Feedback | CFIR Guide Question: Have you/your unit/your organization set goals related to the implementation of the intervention?  Adapted Question: Can you tell me about any goals you, your team, or your organization have set related to the implementation of the National DPP? |
| Implementation Climate - Learning Climate | CFIR Guide Question: To what extent do you feel like you can try new things to improve your work processes?  Adapted Question: To what extent do you feel you have the power to try new methods/processes as they relate to implementing the National DPP? |
| Readiness for Implementation - Leadership Engagement | CFIR Guide Question: What level of involvement has leadership at your organization had so far with the intervention?  Adapted Question: In what ways has leadership at your organization been involved with the implementation of the National DPP lifestyle change program? |
| Readiness for Implementation  - Available Resources | CFIR Guide Question: Do you expect to have sufficient resources to implement and administer the intervention?  Adapted Question: In your experience, what are the key resources your organization needs to implement the National DPP successfully? |
| Readiness for Implementation  - Access to Knowledge & Information | CFIR Guide Question: What kind of training is planned for you? For colleagues?  Adapted Question: Beyond lifestyle coach training, what other kinds of preparation or resources do staff working on the National DPP at your organization receive, if any, to support your ability to implement the lifestyle change program? |
| *Outer Setting* | |
| Patient Needs & Resources | CFIR Guide Question: To what extent were the needs and preferences of the individuals served by your organization considered when deciding to implement the intervention?  Adapted Question: Generally, what has been the response of your participants to the National DPP? How well do you think the National DPP meets the needs of the individuals served by your organization? |
| Cosmopolitanism | CFIR Guide Question: What kind of information exchange do you have with others outside your setting, either related to the intervention, or more generally about your profession?  Adapted Question: What kind of information exchange do you have with others outside of your organization about the National DPP? |
| Peer Pressure | CFIR Guide Question: Can you tell me what you know about any other organizations that have implemented the intervention or other similar programs?  Adapted Question: What do you know about other organizations in your region/state that provide the National DPP lifestyle change program or other similar programs? |
| External Policy & Incentives | CFIR Guide Question: What kind of local, state, or national performance measures, policies, regulations, or guidelines influenced the decision to implement the intervention?  Adapted Question: What kind of local, state, or national policies or initiatives influenced how your organization decided to implement the National DPP? |
